# Representations of recent and remote autobiographical memories in hippocampal subfields

**DOI:** 10.1002/hipo.22155

**Published:** 2013-06-27

**Authors:** Heidi M Bonnici, Martin J Chadwick, Eleanor A Maguire

**Affiliations:** Wellcome Trust Centre for Neuroimaging Institute of Neurology, University College LondonLondon, United Kingdom

**Keywords:** subfields, autobiographical, fMRI, MVPA, consolidation, hippocampus

## Abstract

The hippocampus has long been implicated in supporting autobiographical memories, but little is known about how they are instantiated in hippocampal subfields. Using high-resolution functional magnetic resonance imaging (fMRI) combined with multivoxel pattern analysis we found that it was possible to detect representations of specific autobiographical memories in individual hippocampal subfields. Moreover, while subfields in the anterior hippocampus contained information about both recent (2 weeks old) and remote (10 years old) autobiographical memories, posterior CA3 and DG only contained information about the remote memories. Thus, the hippocampal subfields are differentially involved in the representation of recent and remote autobiographical memories during vivid recall. © 2013 The Authors. Hippocampus Published by Wiley Periodicals, Inc.

There is wide agreement that the hippocampus is necessary for acquiring autobiographical memories, the memories of our personal past experiences, and for their recall in the short-term (Scoville and Milner, 1957). By contrast, there is less consensus about the hippocampal role in recollection of autobiographical memories that are more remote. The standard model of consolidation argues that declarative (including autobiographical) memories become less dependent on the hippocampus over time, eventually abjuring the need for its involvement during retrieval (Marr, 1971; Teyler and DiScenna, 1985; Squire, 1992). Alternative theories, multiple trace theory and scene construction theory, propose instead that the hippocampus is necessary for retrieving vivid autobiographical memories in perpetuity (Nadel and Moscovitch, 1997; Hassabis and Maguire, 2007,2009; Winocur and Moscovitch, 2011). Differential findings across studies of amnesic patients with hippocampal lesions (reviewed in Winocur and Moscovitch, 2011), as well as disparate results from functional magnetic resonance imaging (fMRI) experiments (e.g., Maguire et al., 2001; Ryan et al., 2001; Maguire and Frith, 2003; Gilboa et al., 2004; Piolino et al., 2004; Rekkas and Constable, 2005; Steinvorth et al., 2006; Viard et al., 2007; Watanabe et al., 2012; but see Niki and Luo, 2002; Piefke at al., 2003) contribute to the impasse.

In a recent high-resolution fMRI study, Bonnici et al. (2012a) availed themselves of the opportunity afforded by multivoxel pattern analysis (MVPA; Haynes and Rees, 2006; Norman et al., 2006; Chadwick et al., 2012) to provide an alternative to conventional neuropsychological and fMRI approaches by detecting representations of individual autobiographical memories in patterns of fMRI activity. They examined whether information about specific recent (2 weeks old) and remote (10 years old) autobiographical memories was represented in the hippocampus. They found that information about both types of memory was detectable in the hippocampus, suggesting that it plays a role in the retrieval of vivid autobiographical memories regardless of remoteness. Interestingly, they also reported that while recent and remote memories were both represented within anterior and posterior hippocampus, the latter nevertheless contained more information about remote memories. Thus, the hippocampus respected the distinction between the recent and remote memories.

Functional differentiation down the long axis of the hippocampus has been documented in a range of species including humans (e.g., Moser and Moser, 1998; Maguire et al., 2000; Gilboa et al., 2004; Rekkas and Constable, 2005; Fanselow and Dong, 2010; Poppenk and Moscovitch, 2011; Ranganath and Ritchey, 2012; for a recent review see Poppenk et al., 2013). Bonnici et al.’s (2012a) findings clearly prompt further questions about what might be occurring within anterior and posterior hippocampus during autobiographical memory recall. But there is also another parcellation of the hippocampus that needs to be considered. The hippocampus is composed of a number of subregions CA1, CA2, and CA3 (Lorente de No, 1934), bordered by the dentate gyrus (DG) and subiculum (Amaral and Lavenex, 2007). The findings of Bonnici et al. (2012a) gave no indication as to whether their anterior/posterior differential effects were being driven by all subfields, or by one or two in particular. Studies in rodents and computational models suggest that key computations necessary for memory occur in the subfields, such as pattern separation (in DG and CA3), the process of distinguishing similar memories from each other, and pattern completion (in CA3), which facilitates the retrieval of previously stored memories from partial cues (Marr, 1971; Treves and Rolls, 1994; McClelland et al., 1995; Kesner et al., 2004; Leutgeb et al., 2004,2007; Leutgeb and Leutgeb, 2007; Alvernhe et al., 2008; Hunsaker and Kesner, 2008; Gilbert and Brushfield, 2009; Rolls, 2010; Aimone et al., 2011; O’Reilly et al., 2011). To date, only one study has explored autobiographical memory in relation to the hippocampal subfields. Bartsch et al. (2011) reported that patients with transient global amnesia had apparently focal lesions in CA1 and a concomitant impairment in recalling both recent and remote autobiographical memories. However, focal lesions to other subfields were not examined in this study, so it is unknown whether CA1 is particularly critical for autobiographical memory retrieval, or if a lesion to any subfield would be sufficient to disrupt processing within the hippocampus leading to autobiographical memory recall deficits.

Given the dearth of knowledge about the role of hippocampal subfields in supporting autobiographical memory retrieval, in this study we set out to address three issues that have not been investigated before. First, using high-resolution structural and functional MRI combined with MVPA we sought to ascertain if information about individual autobiographical memories could be detected in specific hippocampal subfields of healthy participants. If so, we aimed to examine whether recent and remote autobiographical memories were differentially represented in those subfields. Third, considering the results of Bonnici et al. (2012a), we also investigated how representations of the memories are related to a subfield’s anterior or posterior hippocampal location.

A prerequisite for our study was the ability to delineate the subfields. We followed a recently published scanning and subfield segmentation protocol that allowed us to manually identify and separate CA1, CA3 (which also included CA2), DG, and subiculum (Bonnici et al., 2012b). This required high-resolution T2-weighted structural MR images acquired on a 3T MRI scanner with an isotropic voxel resolution of 0.5 × 0.5 × 0.5 mm focused on the medial temporal lobes (see Supporting Information for details). Given that sets of these scans were available for the participants in the Bonnici et al. (2012a) study of autobiographical memories, we identified CA1, CA3, DG, and subiculum in each of these participants (Fig. 1), and then reanalyzed the fMRI data from that study, this time focusing our MVPA analyses on the hippocampal subfields.

The participants were 12 healthy right-handed, university-educated subjects (9 female; mean age 27.5 years, SD 3.2, range 22–33). All gave informed written consent to articipation in accordance with the local research ethics committee. Autobiographical memories were elicited 1 week before scanning (see Bonnici et al., 2012a for full details, and also Supporting Information). Recent and remote memories were closely matched on factors such as vividness, level of detail, emotional valence, ease of recall, and frequency of retrieval since the initial episode (see Table S1 in Supporting Information). This was important in order to rule out differences in these basic variables as driving differential effects that might be detected in the fMRI analyses. One week later, participants were scanned using high-resolution (1.5 mm^3^ isotropic voxels) fMRI scanning on a 3T MRI scanner (see Supporting Information for details) while they recalled six autobiographical memories (three recent that were 2 weeks old at the time of interview; 3 weeks old at the time of scanning—mean 13.3 (SD 2.7) days old; 3 remote that were 10 years old—mean 10.4 (SD 0.57) years old).

Participants recalled each memory 14 times in a pseudorandom order, while ensuring that the same memory was not repeated twice or more in a row. On each trial, a verbal cue specified which of the six memories a participant should recall. Following this, an instruction appeared on the screen indicating that participants should close their eyes and vividly recall the cued memory. After 12 s, an auditory tone signalled them to open their eyes. The participant was then required to provide ratings about the preceding recall trial. First, they rated how vivid the memory was in the preceding recall trial (on a scale of 1–5, where 1 was not vivid at all, and 5 was very vivid). Second, they rated how consistently they had recalled it relative to the unfolding of the event as it occurred originally (where 1 was not consistent at all, and 5 was very consistent). These ratings were used to select only the most vivid and most consistently recalled (i.e., ratings of 4 or 5) memories for inclusion in the MVPA analyses, ensuring that we captured genuine re-experiencing. When trials that were not sufficiently vivid or consistent were excluded, this resulted in an average of 11.58 (SD 0.30) trials for each of the three recent memories and an average of 10.14 (SD 0.89) for each of the 3 remote memories, with a mean of 63 (33 recent and 30 remote) trials in total per participant that were entered into the MVPA analysis. After scanning, participants rated on a five-point scale the effort required to recall the memories, where 1 was very easy to recall, and 5 was very difficult to recall. Both recent (mean 1.25, SD 0.32) and remote (1.58, SD 0.54) memories were recalled with ease. They were also asked “Do you feel that repeatedly recalling a memory changed the memory in any way?,” where 1 was not at all, and 5 was very much. Participants indicated that the memories were hardly changed by multiple repetitions (2.08, SD 0.79).

fMRI data were preprocessed using SPM8 (http://www.fil.ion.ucl.ac.uk/spm). We then used a standard MVPA procedure that has been described elsewhere (Chadwick et al., 2010; Bonnici et al., 2012,2012,2012) involving a three-way linear support vector machine (SVM) classifier with 10-fold cross-validation (see Supporting Information for details). A classifier was created for each subfield in each hemisphere. Results for the left and right hemispheres were highly similar, and therefore the data we report here are collapsed across hemispheres. Each classifier was trained on a portion of the fMRI data relating to the three recent autobiographical memories and then tested on an independent set of instances of these memories. This was also the procedure for remote autobiographical memories. This resulted in two accuracy results for each subfield, one for the recent autobiographical memories and one for the remote autobiographical memories.

**Figure 1:**
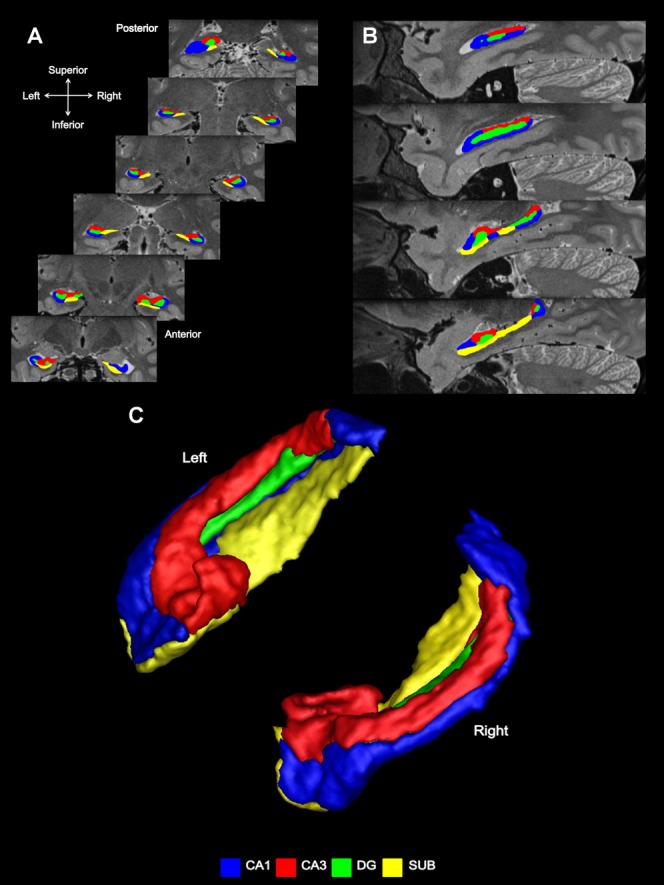
Subfield segmentation. (A) In the coronal plane—coronal sections through an averaged T2-weighted image of the left and right hippocampus of an example participant. (B) Subfield segmentation in the sagittal plane. (C) An example of subfield segmentation in 3D. [Color figure can be viewed in the online issue, which is available at http://wileyonlinelibrary.com.]

We first examined whether it was possible to discriminate between the three recent autobiographical memories from the activity across voxels in each of the four subfields. If information was present in the patterns of fMRI activity that enabled discrimination between the three recent memories, then the classifier would produce a classification result significantly above chance (33%). We found that information was present in CA1 and subiculum which permitted successful detection of the three recent autobiographical memories significantly above chance [CA1: *t*(11) = 3.031, *P* = 0.011; subiculum: *t*(11) = 2.600, *P* = 0.025; Fig. 2, blue line]. This was not the case for CA3 [*t*(11) = 1.513, *P* = 0.158] or DG [*t*(11) = 1.663, *P* = 0.125], where the classifiers’ performance was not significantly different from chance. We then examined the remote memories. In contrast to the recent, we found that the three remote autobiographical memories could be detected significantly above chance in all four subfields [CA1: *t*(11) = 3.786, *P* = 0.003; CA3: *t*(11) = 3.773, *P* = 0.003; DG: *t*(11) = 3.372, *P* = 0.006; subiculum: *t*(11) = 4.227, *P* = 0.001; Fig. 2, red line).

**Figure 2:**
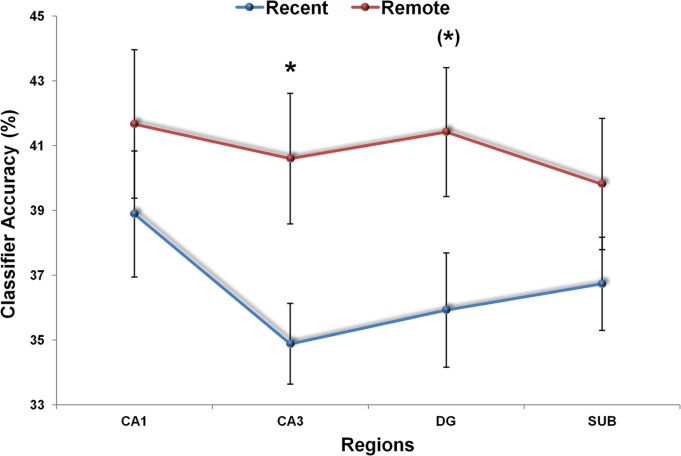
MVPA results for recent and remote autobiographical memories. Recent and remote memories were represented similarly in CA1 and subiculum. Only remote autobiographical memories were detected significantly above chance in CA3 (*P < 0.05), with a similar trend (*) in DG. Error bars represent ±1 standard error of the mean; chance = 33%. [Color figure can be viewed in the online issue, which is available at http://wileyonlinelibrary.com.]

To directly compare recent and remote autobiographical memories, we performed a repeated measure ANOVA. We found a strong trend for the main effect of memory type [*F*(1,11) = 4.211; *P* = 0.065) and a significant interaction between subfield and memory type [*F*(3,33) = 3.092; *P* = 0.04]. Post-hoc *t*-tests revealed that remote autobiographical memories were more readily detected than recent memories in CA3 [*t*(11) = −2.257; *P* = 0.045; Fig. 2). A similar trend was also observed in DG [*t*(11) = −2.009; *P* = 0.07). No significant differences in classifier performance for recent and remote autobiographical memories were apparent for CA1 [*t*(11) = −0.845; *P* = 0.416] or subiculum [*t*(11) = −1.267; *P* = 0.231]. To summarize, we found that it was possible to detect representations of autobiographical memories in individual hippocampal subfields. Moreover, while CA1 and subiculum contained decodable information about both recent and remote autobiographical memories, information about remote more so than recent memories was detectable in CA3 (with a similar trend in DG).

We then divided the hippocampus into anterior and posterior portions based on the protocol of Hackert et al. 2002 (see also Bonnici et al., 2012a), where the anterior 35% of the hippocampus was labeled as anterior and the remainder as posterior (see Supporting Information for mean voxel numbers of each subfield). The end of the uncus was used to delineate the border between the two. MVPA was performed once again, this time on the subfields in the anterior portion (for recent and remote memories), and on the subfields in the posterior portion. There were no significant effects of memory type or subfield in the anterior hippocampal portion (all *F* < 1.99; *P* < 0.285). By contrast, for the posterior portion there was a significant effect of memory type [*F*(1,11) = 7.635; *P* = 0.018] and a significant subfield by memory type interaction [*F*(3,33) = 2.9; *P* = 0.049]. Posthoc investigations revealed that remote autobiographical memories were significantly more detectable than recent memories in CA3 and DG [CA3: *t*(11) = −4.041; *P* = 0.002; DG: *t*(11) = −2.332; *P* = 0.040; CA1: *t*(11) = −1.529; *P* = 0.155; subiculum: *t*(11) = −1.491; *P* = 0.164; Fig. 3).

To summarize, this analysis shows that while all subfields (CA1, CA3, DG, and subiculum) in the anterior hippocampus contained information about both recent and remote autobiographical memories, posterior CA3 and DG only contained decodable information about remote memories. Therefore, while Bonnici et al. (2012a) reported that the hippocampus seems to respect the difference between recent and remote autobiographical memories, our results extend this observation by now showing that it was in particular CA3 and DG that drove this distinction, specifically the portions of these subfields located in the posterior hippocampus. These results therefore resonate with theories that suggest a role for the hippocampus when vividly recollecting autobiographical memories regardless of age (Nadel and Moscovitch, 1997; Hassabis and Maguire, 2007,2009; Winocur and Moscovitch, 2011).

**Figure 3:**
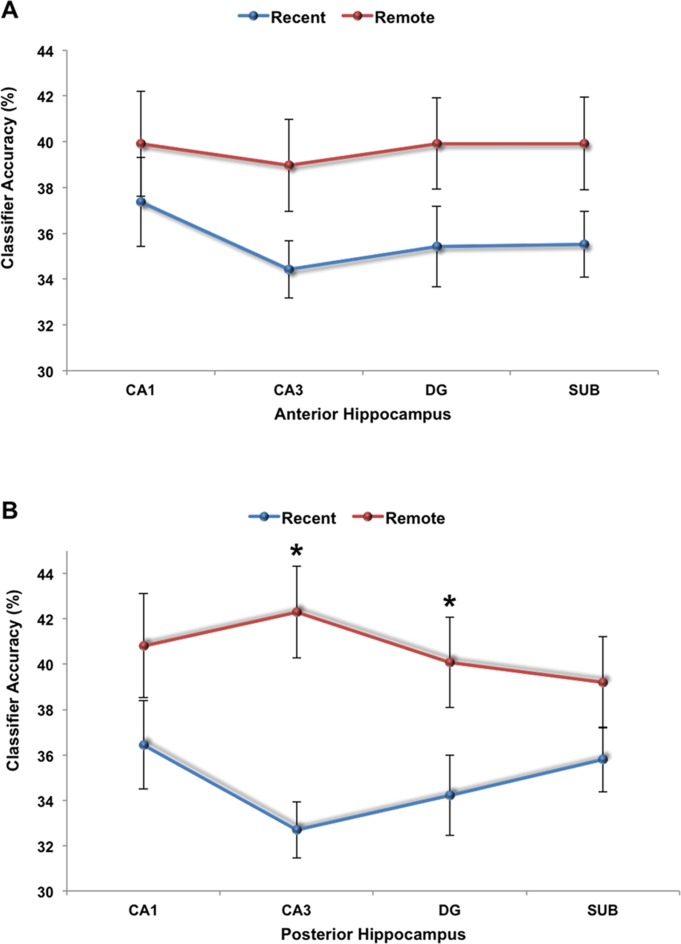
MVPA results for (A) the anterior and (B) the posterior portions of the hippocampus. There were no significant differences in classifier accuracies between recent and remote autobiographical memories in any subfield in the anterior portion. By contrast, for two of the subregions within the posterior hippocampus, CA3 and DG, only remote autobiographical memories were detected significantly above chance (*P < 0.05). Error bars represent ±1 standard error of the mean; chance = 33%. [Color figure can be viewed in the online issue, which is available at http://wileyonlinelibrary.com.]

Perhaps these intrahippocampal distinctions simply reflect qualitative differences between the recent and remote memories. However, the two memory types were highly similar on a range of characteristics that included vividness, ease of recall, and amount of detail (see Supporting Information, Table S1, and Bonnici et al., 2012a for full details of memory matching). Both types of memories were vividly re-experienced suggesting that the remote memories were not more semanticized than the recent memories. Similarly, other factors such as re-encoding, reactivation, or the recall of the prescan interview, which would have affected both recent and remote memories, cannot easily explain the selective findings for remote memories in specifically posterior CA3 and DG.

Considering reasons for our findings, we need to take into account both the posterior hippocampal location of the differential effect for remote memories, and also the selective involvement of CA3 and DG. The posterior hippocampus has been associated with spatial processing (e.g., Moser and Moser, 1998; Maguire et al., 2000). Bonnici et al. (2012a) suggested that the posterior hippocampus may implement the spatial framework for scenes into which the elements of a memory are reconstructed (Hassabis and Maguire, 2007,2009), in line with findings from patients with hippocampal damage who have lost the ability to construct spatially coherent scenes (e.g., Hassabis et al., 2007; Race et al., 2011; Mullally et al., 2012—but see Squire et al., 2010 and Maguire and Hassabis, 2011 for a response). Bonnici et al. (2012a) further speculated that recent memories may be experienced as coherent scenes or events that are temporarily represented in the hippocampus (utilizing anterior and posterior aspects), with neocortical consolidation happening relatively quickly. The constituent elements of autobiographical memories are then the preserve of the neocortex. At retrieval, this piecemeal information is automatically funneled back into the hippocampus, but in order to be assembled into a coherent form; this requires the scene construction process that takes place in the posterior hippocampus. They suggest this is why remote memories were discernible to a greater degree in posterior hippocampus, because they rely on this process more than do recent memories.

By contrast, CA3 and DG are linked with pattern separation and CA3 with pattern completion (Marr, 1971; Treves and Rolls, 1994; McClelland et al., 1995; Kesner et al., 2004; Leutgeb et al., 2004; Leutgeb and Leutgeb, 2007; Leutgeb et al., 2007; Alvernhe et al., 2008; Hunsaker and Kesner, 2008; Gilbert and Brushfield, 2009; Aimone et al., 2011; O’Reilly et al., 2011). We hypothesize that if remote autobiographical memories have to undergo more reconstruction than recent memories, then the accumulation of memory elements and spatial contexts in posterior hippocampus might trigger CA3-mediated pattern completion to a greater extent. Clearly this is speculative, and additional studies are required to explore this further, as well as to establish precisely what each of the subfields do, both anteriorly and posteriorly, and the functional connectivity between them. The high-resolution structural and functional MRI approach adopted here, and the ability to separate the hippocampal subfields, demonstrates that these kinds of questions are now tractable, presenting new opportunities to examine how autobiographical memories are processed and represented at this fundamental level.
