# Supplementary material for: Representations of recent and remote autobiographical memories in hippocampal subfields
Source: Hippocampus. 2013 Jun 27;23(10):849–54. doi: 10.1002/hipo.22155 (PMC4281962; doi:10.1002/hipo.22155)
Supplement: Supplementary file 1 [file hipo-23-849-sd1.docx]

**Representations of recent and remote autobiographical memories in hippocampal subfields**

Heidi M. Bonnici, Martin J. Chadwick and Eleanor A. Maguire

**Supporting Online Material**

**Materials and Methods**

The methodological details of this study are described in full in Bonnici et al. (2012a). Key details are reprised here for convenience.

**Pre-scan interview**

The interview technique employed in this experiment was a standard method used in numerous previous studies (e.g. Maguire et al., 2001; Addis et al., 2004a,b; Summerfield et al., 2009). One week prior to scanning, participants were asked to recollect events that happened from a particular time frame (two weeks ago or ten years ago). An example of the type of memory that was required was provided and it was emphasised that very private or emotional memories, events that happened repeatedly or were very similar to other events, or memories related to public events were not suitable. The memories should unfold in an event-like way, and be very clear and vivid such that when recollecting the memory they felt as if they were re-experiencing the event. Participants were also instructed that they should provide memories that they had rarely thought about since the time the original event had occurred. General probes were given by the interviewer when required (e.g. ‘what else can you tell me about this event’). Notes were taken about each memory by the interviewer. Having described a memory, participants then rated each memory along a range of parameters (see Table S1).

| **Table S1: Memory characteristics** |  |  |  |  |
| --- | --- | --- | --- | --- |
| **Variable** | **Recent** | **Remote** | **Recent vs Remote** | |
|  | **mean (SD)** | **mean (SD)** | **t value** | **p value** |
| Recall frequency before the interview | 1.64 (0.611) | 1.83 (0.415) | 1.258 | 0.235 |
| Recall frequency between the interview and scan | 1.08 (0.208) | 1.03 (0.095) | 1.483 | 0.166 |
| Vividness | 4.58 (0.352) | 4.39 (0.372) | 1.549 | 0.15 |
| Level of detail | 4.47 (0.414) | 4.14 (0.576) | 1.7 | 0.117 |
| 1st/3rd person perspective | 1 (0) | 1.08 (0.149) | 1.915 | 0.082 |
| Emotional valence | 3.17 (0.301) | 3.14 (0.172) | 0.372 | 0.717 |
| Active/static event | 1 (0) | 1.03 (0.095) | 1 | 0.339 |
| Consistency of recall trial-to-trial | 4.83 (0.225) | 4.72 (0.372) | 1.317 | 0.215 |
| Ratings were on a scale of 1 to 5, where 1 was the minimum and 5 the maximum. For emotional valence: 1,2 = negative, 3 = neutral, 4,5 = positive. For 1st/3rd person perspective: 1 = 1st person, 2 = 3rd person. For active/static event: 1 = active, 2 = static. | | | | |

During the interview, participants generally recalled 6 to 7 memories from each time period. Based on the ratings for these memories, six memories (three recent and three remote) were then selected from this memory pool to be used in the scan experiment. Several criteria guided the selection of the memories for inclusion. Only those memories that had very high ratings for variables such as vividness (see Table 1), and that were matched to each other both within the recent and remote sets and between the two sets across all the variables, were included. In addition, the experienced interviewer had to be satisfied that the memories were richly detailed and vivid, and seemed to be genuinely re-experienced by the participant. The recent memories were on average 13.3 (SD 2.7) days old, while the remote memories were on average 10.4 (SD 0.57) years old (note that memories were seven days older when scanned a week later). Mean ratings for these memories are shown on Table 1, and confirm that the memories were vivid and could be recalled consistently on repeated occasions. Of note, the memories were also rated as not having been recalled very much since the initial occurrence of the event. Statistical comparisons (two tailed t-tests) between recent and remote memories (also reported on Table 1) showed there were no significant differences between the two types of memory for any of the variables.

The interview material was subjected to a careful review to look for clues that might betray differences between the recent and remote memories used in the scanning experiment, but nothing was found. For instance, we conducted a separate behavioural experiment where 27 naïve participants were given the memory cues from the study and were asked to decide whether a memory was recent or remote based on the cues. An ANOVA comparing the actual memory labels with the participants’ labels showed no significant differences, F(1,69)=2.33; p=0.13). In addition, the memories were coded for the number of overlapping events, locations and people, in case any biases were present: means for recent memories - events: 0; locations: 0; people: 0.4; means for remote memories – events: 0; locations: 0; people: 0.5. It is clear that the amount of inter-memory similarity was very low, and did not differ between the recent and remote memories.

**MRI scanning**

We acquired high resolution fMRI data in a limited volume focused on the medial temporal lobes, using a 3T Magnetom Allegra head only MRI scanner (Siemens Healthcare, Erlangen, Germany) operated with the standard transmit-receive head coil and a T2*-weighted single-shot echo-planar imaging (EPI) sequence in a single session (in-plane resolution = 1.5 x 1.5 mm²; matrix = 128 x 128; field of view = 192 x 192 mm²; 35 slices acquired in interleaved order; slice thickness = 1.5mm with no gap between slices; echo time TE = 30ms; asymmetric echo shifted forward by 26 phase-encoding (PE) lines; echo spacing = 560 µs; repetition time TR = 3.5s; flip angle α = 90°). All data were acquired at 0° angle in the anterior-posterior axis. An isotropic voxel size of 1.5 x 1.5 x 1.5 mm was chosen for an optimal trade-off between BOLD sensitivity and spatial resolution. Further, the isotropic voxel dimension reduced re-sampling artefacts when applying motion correction. To ensure optimal data quality, images were reconstructed online and underwent online quality assurance ([Weiskopf et al., 2007](#_ENREF_65)). For distortion correction ([Hutton et al., 2002](#_ENREF_28)), field maps were acquired with the standard manufacturer’s double echo gradient echo field map sequence (TE = 10.0 and 12.46 ms, TR 1020ms; matrix size, 64x64), using 64 slices covering the whole head (voxel size 3 x 3 x 3 mm). In addition to the functional scans, a whole brain T1-weighted 3D FLASH sequence was acquired with a resolution of 1 x 1 x 1 mm.

High-resolution structural images were acquired in a limited volume focused on the medial temporal lobes on a 3T whole body MRI scanner (Magnetom TIM Trio, Siemens Healthcare, Erlangen, Germany) operated with the standard transmit body coil and 32-channel head receive coil. A single-slab 3D T2-weighted turbo spin echo sequence with variable flip angles (SPACE, [Mugler et al., 2000](#_ENREF_38)) in combination with parallel imaging was employed to simultaneously achieve a high image resolution of ~500 µm, high sampling efficiency and short scan time while maintaining a sufficient signal-to-noise ratio (SNR). After excitation of a single axial slab the image was read out with the following parameters: resolution = 0.52 x 0.52 x 0.5 mm^3^, matrix = 384 x 328, partitions = 104, partition thickness = 0.5 mm, partition oversampling = 15.4%, field of view = 200 x 171 mm^2^, TE = 353 ms, TR = 3200 ms, GRAPPA x 2 in phase-encoding (PE) direction, bandwidth = 434 Hz/pixel, echo spacing = 4.98 ms, turbo factor in PE direction = 177, echo train duration = 881, averages = 1.9. For reduction of signal bias due to, for example, spatial variation in coil sensitivity profiles, the images were normalized using a prescan and a weak intensity filter was applied as implemented by the scanner’s manufacturer. To improve the SNR of the anatomical image, four scans were acquired for each participant, coregistered and averaged.

**Delineating the hippocampal subfields**

Manual segmentation of the subfields was performed using the protocol of Bonnici et al. (2012b) on the averaged T2 high-resolution (0.5mm^3^) structural images of each participant. This resulted in identification of CA1, CA3, DG and subiculum for each participant in each hemisphere. The average amount of time taken to segment the subfields of one hippocampus was approximately two days. Intra-rater reliability was calculated using the Dice overlap metric (Dice, 1945), defined as the volume of overlap between two regions of interest, divided by the mean volume. As in other subfield segmentation studies (Van Leemput et al., 2008; Yushkevich et al., 2009; Malykhin et al., 2010), five consecutive slices located in the body of the hippocampus were chosen. Intra-rater reliability was assessed by comparing two sets of segmentations performed by HMB with a 6 month interval between segmentations: CA1 0.80, CA3 0.77, DG 0.74, subiculum 0.82. The mean number of (1.5mm^3^) voxels in each subfield was: *whole hippocampus* – CA1 493.38 (68.76), CA3 299.04 (32.47), DG 201.67 (21.58), subiculum 227.38 (32.97); *anterior portion* – CA1 232.88 (42.38), CA3 158.38 (24.85), DG 125.33 (16.05), subiculum 93.42 (19.10); *posterior portion* – CA1 308.29 (34.49), CA3 158.21 (25.45), DG 139.38 (19.57), subiculum 139.04 (24.98).

**Image preprocessing**

Image pre-processing was performed using SPM8 (http://www.fil.ion.ucl.ac.uk/spm). The first six EPI volumes were discarded to allow for T1 equilibration effects ([Frackowiak et al., 2004](#_ENREF_16)). The remaining EPI images were then realigned to correct for motion effects, and minimally smoothed with a 3mm FWHM Gaussian kernel. A linear detrend was run on the images to remove any noise due to scanner drift ([LaConte et al., 2005](#_ENREF_31)) using customised matlab code. Next the data were convolved with the canonical hemodynamic response function (HRF) to increase the signal-to-noise ratio ([Frackowiak et al., 2004](#_ENREF_16)). This HRF convolution effectively doubled the natural BOLD signal delay, giving a total delay of approximately 12s. To compensate for this delay, all onset times were shifted forward in time by three volumes, yielding the best approximation to the 12s delay given a TR of 3.5s and rounding to the nearest volume. Analysis focused on the 12 second periods of vivid recall giving a total of four functional volumes per trial.

**MVPA**

*Overview:* A support vector machine (SVM) classifier was created for each subfield. Each classifier was trained on a portion of the fMRI data relating to the three recent memories and then tested on an independent set of instances of these memories. This was also the procedure for remote memories. This resulted in two accuracy results for each subfield, one for the recent memories and one for the remote memories.

*Procedure:* We used a standard MVPA procedure that has been described in detail elsewhere (Chadwick et al., 2010, 2012; Bonnici et al., 2012a,b,c). To reprise briefly, the overall classification procedure involved splitting the fMRI data into two segments: a “training” set used to train a classifier with fixed regularization hyperparameter C = 1, in order to identify response patterns related to the memories being discriminated, and a “test” set used to independently test the classification performance (Duda et al., 2001), using a ten-fold cross-validation procedure. Prior to multivariate classification, feature selection (Guyon and Elisseeff, 2003) was performed on the data from the training set (thereby ensuring that this step was fully independent from final classification, which is critical for avoiding “double-dipping”, Kriegeskorte et al., 2009). This was conducted using a standard multivariate searchlight strategy within a region of interest. For a given voxel, we first defined a small sphere with a radius of three voxels centred on the given voxel (Kriegeskorte et al., 2006; see also Hassabis et al., 2009; Chadwick et al., 2010, 2012; Bonnici et al., 2012a,c). Note that the spheres were restricted so that only voxels falling within the given region of interest were included. Therefore, the shape of the sphere and the number of voxels within it varied depending on the proximity to the region of interest’s borders. This procedure then allowed the selection of the searchlight voxel set that contained the greatest degree of decoding information within the training dataset. Using this voxel subset, the SVM classifier was trained to discriminate between, for example, the three recent memories using the “training” image dataset, and tested on the completely independent “test” dataset. The classification was performed using the LIBSVM implementation (Chang and Lin, 2011).

Standard SVMs are binary classifiers that operate on two-class discrimination problems, whereas our data involved a three-class problem (i.e. three recent memories or three remote memories). The SVM can, however, be arbitrarily extended to work in cases where there are more than two classes. Typically this is done by reducing the single multiclass problem into multiple binary classification problems that can be solved separately and then recombined to provide the final class prediction (Allwein et al., 2000). We used the well-established Error Correcting Output Codes approach (Dietterich and Bakiri, 1994) and computing of the Hamming distance (Hamming, 1950) as described in detail elsewhere (Hassabis et al., 2009; Chadwick et al., 2010).

**Data analysis**

The classifier accuracy values for each subfield were compared to chance. Given that we were only interested in whether results were significantly above chance, one tailed t-tests were used. Other comparisons were conducted using repeated measures ANOVAs and significant results were subsequently interrogated using two-tailed paired t-tests. A threshold of p<0.05 was employed throughout.

**Results**

In another set of analyses, we collapsed across individual memories and examined whether it was possible to in general distinguish recent from remote memories. Table S2 below summarises the results, for each entire subfield (‘whole’), and the anterior (‘ant’) and posterior (‘post’) segments of each subfield. In line with previous findings reported by Bonnici et al. (2012a), we found that classifiers operating in each hippocampal subregion could classify recent and remote autobiographical memories significantly above chance (which was 50%).

**Table S2: Recent versus remote memory decoding**

Region t-value df sig(2-tailed) sig(1-tailed)

| CA1whole | 3.396 | 11 | .006 | .003 |
| --- | --- | --- | --- | --- |
| CA3whole | 3.120 | 11 | .010 | .005 |
| DGwhole | 3.906 | 11 | .002 | .001 |
| SUBwhole | 3.229 | 11 | .008 | .004 |
| CA1ant | 4.017 | 11 | .002 | .001 |
| CA3ant | 4.029 | 11 | .002 | .001 |
| DGant | 3.301 | 11 | .007 | .004 |
| SUBant | 3.005 | 11 | .012 | .001 |
| CA1post | 2.465 | 11 | .031 | .002 |
| CA3post | 2.799 | 11 | .017 | .009 |
| DGpost | 2.648 | 11 | .023 | .011 |
| SUBpost | 2.859 | 11 | .016 | .008 |

**References**

Addis DR, Moscovitch M, Crawley AP, McAndrews MP. 2004a. Recollective qualities modulate hippocampal activation during autobiographical memory retrieval. Hippocampus 14: 752-762.

Addis DR, McIntosh AR, Moscovitch M, Crawley AP, McAndrews MP. 2004b. Characterizing spatial and temporal features of autobiographical memory retrieval networks: a partial least squares approach. NeuroImage 23: 1460-1471.

Allwein E, Shapire R, Singer Y. 2000. Reducing multiclass to binary: a unifying approach for margin classifiers. J Machine Learn Res 1: 113–141.

Bonnici HM, Chadwick MJ, Lutti A, Weiskopf N, Maguire EA. 2012a. Detecting representations of recent and remote autobiographical memories in vmPFC and hippocampus. J Neurosci 32: 16982-16991.

Bonnici HM, Chadwick MJ, Kumaran D, Hassabis D, Weiskopf N, Maguire EA. 2012b. Multi-voxel pattern analysis in human hippocampal subfields. Fron Hum Neurosci 6: 290.

Bonnici HM, Kumaran D, Chadwick MJ, Weiskopf N, Hassabis D, Maguire EA. 2012c. Decoding representations of scenes in the medial temporal lobes. Hippocampus 22: 1143-1153.

Chadwick MJ, Bonnici HM, Maguire EA. 2012. Decoding information in the human hippocampus: A user's guide. Neuropsychologia 50: 3107-3121.

Chadwick MJ, Hassabis D, Weiskopf N, Maguire EA. 2010. Decoding individual episodic memory traces in the human hippocampus. Curr Biol 20: 544-547.

Chang C, Lin C. 2011. LIBSVM: A library for support vector machines. AVM Transactions on Intelligent Systems 2: Article 17.

Dice LR. 1945. Measures of the amount of ecologic association between species. Ecology 26: 297-302.

Dietterich TD, Bakiri G. 1994. Solving multiclass learning problems via error-correcting output codes. J Artificial Intell *Res* 2: 263–286.

Duda OR, Hart PE, Stork DG. 2001. Pattern Classification. New York: Wiley.

Frackowiak RSJ, Friston KJ, Frith CD, Dolan RJ, Price CJ, Zeki S, Ashburner JT, Penny WD. 2004. Human Brain Function*.* New York: Elsevier Academic Press.

Guyon I, Elisseeff A. 2003. An introduction to variable and feature selection. J Mach Learn Res 3: 1157-1182.

Hamming RW. 1950. Error-detecting and error-correcting. Bell System Technical Journal 29: 147–160.

Hassabis D, Chu C, Rees G, Weiskopf N, Molyneux PD, Maguire EA. 2009. Decoding neuronal ensembles in the human hippocampus. Curr Biol 19: 546-554.

Hutton C, Bork A, Josephs O, Deichmann R, Ashburner J, Turner R. 2002. Image distortion correction in fMRI: A quantitative evaluation. Neuroimage 16: 217-240.

Kriegeskorte N, SimmonsWK, Bellgowan PSF, Baker CI. 2009. Circular analysis in systems neuroscience: the dangers of double dipping. Nat Neurosci 12: 535–540.

Kriegeskorte N, Goebel R, Bandettini P. 2006. Information-based functional brain mapping. Proc Natl Acad Sci USA 103: 3863-3868.

LaConte S, Strother S, Cherkassky V, Anderson J, Hu X. 2005. Support vector machines for temporal classification of block design fMRI data. Neuroimage 26: 317-329.

Maguire EA, Henson RN, Mummery CJ, Frith CD. 2001. Activity in prefrontal cortex, not hippocampus, varies parametrically with the increasing remoteness of memories. Neuroreport 12: 441-444.

Malykhin NV, Lebel RM, Coupland NJ, Wilman AH, Carter R. 2010. In vivo quantification of hippocampal subfields using 4.7 T fast spin echo imaging. NeuroImage 49: 1224-1230.

Mugler JP, 3rd, Bao S, Mulkern RV, Guttmann CR, Robertson RL, Jolesz FA, Brookeman JR. 2000. Optimized single-slab three-dimensional spin-echo MR imaging of the brain. Radiology 216: 891-899.

Summerfield JJ, Hassabis D, Maguire EA. 2009. Cortical midline involvement in autobiographical memory. NeuroImage 44: 1188-1200.

Van Leemput K, Bakkour A, Benner T, Wiggins G, Wald LL, Augustinack J, Dickerson BC, Golland P, Fischl B. 2008. Model-based segmentation of hippocampal subfields in ultra-high resolution in vivo MRI*.* International Conference on Medical Image Computing and Computer-Assisted Intervention 11: 235-243.

Weiskopf N*,* Sitaram R, Josephs O, Veit R, Scharnowski F, Goebel R, Birbaumer N, Deichmann R, Mathiak K. 2007. Real-time functional magnetic resonance imaging: methods and applications. Magn Reson Imaging 25: 989-1003.

Yushkevich PA, Avants BB, Pluta J, Das S, Minkoff D, Mechanic-Hamilton D, Glynn S, Pickup S, Liu W, Gee JC, Grossman M, Detre JA. 2009. A high-resolution computational atlas of the human hippocampus from postmortem magnetic resonance imaging at 9.4 T. NeuroImage 44: 385-398.
